# Patterns of Tobacco Use and Dual Use in US Young Adults: The Missing Link between Youth Prevention and Adult Cessation

**DOI:** 10.1155/2012/679134

**Published:** 2012-05-14

**Authors:** Jessica M. Rath, Andrea C. Villanti, David B. Abrams, Donna M. Vallone

**Affiliations:** ^1^Department of Research and Evaluation, Legacy, Washington, DC 20036, USA; ^2^The Schroeder Institute for Tobacco Research and Policy Studies, Legacy, Washington, DC 20036, USA; ^3^Department of Health, Behavior and Society, Johns Hopkins Bloomberg School of Public Health, Baltimore, MD 21205, USA; ^4^Department of Oncology, Georgetown University Medical Center and Lombardi Comprehensive Cancer Center, Washington, DC 20007, USA

## Abstract

Few studies address the developmental transition from youth tobacco use uptake to regular adulthood use, especially for noncigarette tobacco products. The current study uses online panel data from the Legacy Young Adult Cohort Study to describe the prevalence of cigarette, other tobacco product, and dual use in a nationally representative sample of young adults aged 18–34 (*N* = 4,201). Of the 23% of young adults who were current tobacco users, 30% reported dual use. Ever use, first product used, and current use were highest for cigarettes, cigars, little cigars, and hookah. Thirty-two percent of ever tobacco users reported tobacco product initiation after the age of 18 and 39% of regular users reported progressing to regular use during young adulthood. This study highlights the need for improved monitoring of polytobacco use across the life course and developing tailored efforts for young adults to prevent progression and further reduce overall population prevalence.

## 1. Introduction

In 2010, young adults aged 18–25 reported the highest prevalence of current use of a tobacco product (40.8%) compared to youth (ages 12–17) or adults (ages 26 and older) [1]. Although young adult (aged 18–24) cigarette smoking prevalence decreased overall (24.4% to 20.1%) from 2005 through 2010 [2], the highest prevalence of smoking among all adults was reported among this age segment in 2005 and 2006 [3, 4]. Since the Master Settlement Agreement, which restricted tobacco marketing to youth [5], young adults have become an increasingly important target audience for tobacco industry attention [6]. Young adulthood marks an important developmental period for leaving home and school, increased stress and pressure, identity exploration, and the establishment of health behaviors that will persist throughout adulthood [7]. It has also been shown to be a particularly salient time for progression to regular tobacco use [8]. The transition from youth smoking initiation (and its primary prevention) to adult established smoker (and cessation treatment interventions) is an understudied developmental period along the trajectories and pathways of progression to regular tobacco use, nicotine dependence, and difficulty quitting [9, 10]. Understanding the role tobacco use behavior plays during this critical life stage can offer important opportunities to significantly reduce tobacco use prevalence and its preventable harms.

Several studies indicate that this age group is also at increased risk for using other noncigarette tobacco products. The National College Health Assessment survey (NCHA-II) reported that 14.8% of college students used cigarettes in the past 30 days, 7.8% used cigars, little cigars, or clove cigarettes in the past 30 days, and 3.9% used smokeless tobacco [11]. Research also highlights the prevalence of hookah use in the young adult population, particularly among college students [11–15]. For example, more than a quarter of college students have smoked tobacco from a hookah or water pipe, with 8.5% reporting past 30-day use [11]. In 2007, an estimated 200–300 hookah cafés/bars operated in the USA, usually near college campuses, with more appearing every day [16]. Although limited data is available on the trial of snus, young adults indicate a high level of interest in these products [17]. New electronic nicotine delivery devices (ENDS), erroneously called electronic or e-cigarettes, may also be especially appealing to young adults, providing aerosolized doses of nicotine with appealing flavors [18]. Two recent studies also reported on use of electronic cigarettes, e-cigarettes, or electronic nicotine delivery systems (ENDS) in the young adult population; one study showed the highest prevalence of ever use in 18–24 year olds at 10.1% [19] and the other suggests an inverse relationship between use of ENDS and age, with higher use among younger adults [20]. Additionally, rates of dual use and polytobacco use in the young adult population are of increasing concern. In a nationally-representative sample, young adults aged 18–24 reported the highest prevalence of polytobacco use, defined as concurrent use of more than one tobacco product, compared to those adults ≥25 years [21]. In Minnesota, more than 24% of young adult current cigarette smokers reported current use of other non-cigarette products [22], and in a Canadian sample, more than 26% of young adults reported lifetime polytobacco use [23].

Since 1992, smoking patterns for young adults have shifted to reflect an increase in light and intermittent smoking [24]. However, tobacco use surveillance measures have not been modified to detect these changes in tobacco use behavior. A recent study by Foldes et al. [22] demonstrated that using the adolescent measure of current smoking (i.e., have you smoked a cigarette in the past 30 days?) resulted in a 7% increase in smoking prevalence among young adults, 18.7% of which were considered previously unrecognized smokers. Twenty-eight percent of these previously unrecognized light and intermittent smokers reported initiating smoking after age 18 years and 35.5% reported starting to smoke regularly between the ages of 18 and 24 [22].

In a rapidly changing landscape of tobacco use patterns across an increasingly diversified offering of tobacco products, the need for rapid and reliable surveillance is even more critical. The passage of the 2009 FDA Family Smoking Prevention and Tobacco Control Act (FSPTCA) provides a new set of regulatory tools to reduce the harms of tobacco use [25]. The new FDA regulation has also coincided with the introduction of a number of new products to deliver nicotine to the human brain (e.g., snus, dissolvables, and e-cigarettes) that may be especially attractive to youth and young adults [17, 18, 20]. All major cigarette companies worldwide are positioning themselves in the market for snus, the Swedish name for snuff. Most of the new products are smokeless, spitless, low nitrosamine tobacco and use existing major cigarette brand names to market the products [26–28]. Companies are using advertising such as “Fits Alongside Your Smokes” to promote these products for dual use [29, 30]. Moreover, it is likely that new innovations of ENDS will be marketed in the near future [18, 31]. Thus, it is even more imperative that surveillance of young adults keep up with and measure changing trends as rapidly and rigorously as possible to serve as an early warning tool (i.e., the “canary in the coal mine”) for regulators and policymakers. The current study uses data from a large, nationally representative sample of young adults to describe prevalence, patterns, and predictors of cigarette, other tobacco product, and dual use in this population.

## 2. Methods

### 2.1. Participants

The Legacy Young Adult Cohort Study is designed to understand the trajectories of tobacco use in a young adult population using a longitudinal cohort sample (*N* = 4, 215). The 18–34-year age range was selected in order to be consistent with other Legacy research. For example, previous publications by the Legacy research group demonstrate differences between younger (18–24) and older (25–34) young adults [32]. Baseline data from the cohort were used to estimate prevalence of cigarette and other tobacco product use in this nationally representative sample of young adults aged 18–34 drawn from the Knowledge Networks' KnowledgePanel^®^. KnowledgePanel^®^ is a commercial online panel of adults aged 18 and older that covers both the online and offline populations in the U.S (http://www.knowledgenetworks.com/knpanel/index.html). The cohort was recruited via address-based sampling, a probability-based random sampling method which provides statistically valid representation of the USA population, including cell-phone-only households, African Americans, Latinos, and younger adults. Knowledge Networks also provides households without internet access with a free netbook computer and internet service to reduce response bias in typical online survey samples. The baseline survey was fielded for one month in the summer of 2011 and African American and Hispanic respondents were oversampled to ensure sufficient samples for subgroup analysis. The household recruitment rate for this study was 14.8% and, in 65% of these households, one member completed a survey. For this particular study, only one panel member per household was selected at random to be part of the study sample and no members outside the panel were recruited. The study completion rate was 56.9% and thus, the cumulative response rate was 5.5%. Appendix A provides a demographic comparison of panel members to the overall USA population aged 18–34 and demonstrates the representativeness of the Knowledge Networks sample (see Supplementary Material available online at doi:10.1155/2012/679134). Poststratification adjustments were used to offset any nonresponse or noncoverage bias by weighting the data. Observations were deleted for those respondents where data was missing on the item which assessed ever tobacco use (*N* = 14). This study was approved by the Independent Investigational Review Board, Inc. Immediately upon completion of the survey, points were awarded to each respondent. This survey incentive was 10,000 points which is the equivalent of $10. When panel respondents reach 25,000 points by completing numerous surveys, they receive a check for $25.

### 2.2. Measures

Demographic items included age (grouped as 18–24 and 25–34), gender, and race/ethnicity (White, non-Hispanic; Black, non-Hispanic; other, non-Hispanic; and Hispanic). Educational attainment (less than high school, high school, some college, Bachelor's degree, and graduate or professional degree), current employment status, and self-described financial situation (live comfortably, meet needs with a little left, just meet basic expenses, and do not meet basic expenses) were also included.

Tobacco use was assessed with measures of ever tobacco use, first tobacco product tried, past 30-day use, every day or someday use, and number of cigarettes smoked per day for each day of the week. For ever use, first product tried, and past 30-day use, response categories included cigarettes, cigars, pipe (with tobacco), little cigars/cigarillos/bidis (like Black & Milds, Swisher Sweets, Phillies Blunt, or Captain Black), e-cigarettes (like BLU or NJOY), chewing tobacco (like Levi Garrett, Red Man, or Beech Nut), dip/snuff (like Skoal or Copenhagen), snus (like Camel Snus), dissolvable tobacco products (like Ariva, Stonewall, Camel Orbs, Sticks or Strips), and hookah/shisha (hookah tobacco); for first product tried, participants were able to fill in an “other” category. Ever use and first product used also captured consumption of nicotine replacement products (like gum, patches, lozenges). Participants were asked to recall their age at tobacco product initiation and at progression to regular use, defined as monthly use. Given the rising prevalence of hookah use, participants were also asked whether they had ever visited a hookah bar or restaurant.

Tobacco use was categorized as respondents who reported current “every day” or “some days” use of cigarettes or tobacco products. Categories included “cigarettes only,” “cigarettes and other tobacco products,” and “other tobacco products only.” Individuals who reported no current tobacco product use, including those who never used a product, were classified as “neither.” Individuals who reported using both cigarettes and other tobacco products “every day” or “some days” were classified as dual users.

### 2.3. Data Analysis

All analyses were performed using Stata IC 11.0 [33] and data were weighted to produce nationally representative prevalence estimates. Univariate analyses were conducted to describe the distribution of sociodemographic variables and bivariate analyses estimated the prevalence of tobacco use by product and the prevalence of dual use across sociodemographic and socioeconomic variables. Differences in means or prevalence estimates were assessed by nonoverlapping 95% confidence intervals and *P* values were estimated using the design-based *F* statistic. Multinomial multivariate logistic regression models were used to calculate the adjusted relative risk ratios (RRRs) in Table 3 for current cigarette-only use, dual use, and other tobacco-product-only use compared to no tobacco use for all covariates in the model accounting for survey weights.

## 3. Results

 Participants ranged in age from 18 to 34 years (*N* = 4201), 50% were males (CI: 48%–52%), and 50% were females (CI: 48%–52%). Sixty percent of the population was White (CI: 58%–62%), 13% Black (CI: 12%–15%), 7% other (CI: 6%–9%), and 19% Hispanic (CI: 18%–21%). The majority of participants (43%) had some college education (CI: 41%–45%) while 16% had a Bachelor's degree (CI: 15%–18%), 7% had a graduate degree (CI: 6%–8%), and 34% had a high school education or less (CI: 32%–36%). The majority of the sample works full time (47%; CI: 45%–49%), 22% work part time (CI: 20%–23%), and 32% does not currently work for pay (CI: 30%–34%). Financial situation was assessed by the following categories: live comfortably (23%; CI: 21%–25%), meet needs with a little left (35%; CI: 33%–37%), just meet basic expenses (32%; CI: 30%–34%), and do not meet basic expenses (9%; CI: 8%–11%).

More than half of the sample had ever smoked cigarettes (51%), 31% had ever smoked cigars, and 26% had ever smoked little cigars/cigarillos/bidis (Table 1). First product used followed the same order: 73% initiated with cigarettes, 11% with cigars, 5% with little cigars/cigarillos/bidis, and 4% with hookah. Of those who reported every day or someday smoking, 87% had smoked in the past 30 days (mean number of days of cigarette use in the past 30 = 23 days), 19% currently smoke cigars (mean = 6 days of past 30), and 16% currently smoke little cigars/cigarillos/bidis (mean = 11 days of past 30). In addition, 8% of persons reporting every day or someday use of cigarettes or other tobacco products reported hookah use in the past 30 days (17% ever use of hookah), with a mean of 7 hookah uses in the past 30 days. Ever use and current use of e-cigarettes, chewing tobacco, pipes, dip, snus, dissolvable products, and nicotine products were all at 10% or less (Table 1). Twenty-three percent of the full sample reported current use of cigarettes and/or other tobacco products, with 7% reporting dual use. This corresponds to a 30% prevalence of dual use among current tobacco users.

Bivariate correlations were assessed between selected demographics and current tobacco product use (Table 2). There were no statistically significant differences in tobacco product use among those aged 18–24 years versus those aged 25–34 years. Females were significantly less likely than males to use cigarettes and other tobacco products (5% versus 9%; *P* < .001) as well as other tobacco products only (1% versus 6%; *P* < .001). A significantly higher proportion of Hispanics reported use of neither cigarettes or other tobacco products (83% versus 75%; *P* = .017), compared to Whites and Black participants were significantly less likely to use other tobacco products only compared to Whites (2% versus 4%; *P* = .017). Participants with at least some college education, compared to high school education or less, were significantly more likely to be nonsmokers and nonusers of other tobacco products with 93% of those with a graduate or professional degree not using tobacco products versus 68% of participants with high school educations only (*P* < .001). Twenty-three percent of persons reporting that they do not meet their basic expenses are cigarette smokers and 12% use cigarettes and other tobacco products. This is significantly different than those reporting living comfortably (5% smokers; 4% smoking and using other tobacco products; *P* < .001).

In the group of dual users, the highest prevalence of past 30-day use was reported for the following products: cigarettes (98%), cigars (23%), little cigars (26%), hookah (17%), dip or snuff (12%), chewing tobacco (12%), and e-cigarettes (9%). Past 30-day use of snus in this group was 7% and dissolvable tobacco product use was 3%. Individuals who reported using cigarettes only had a mean daily use of 9.20 cigarettes per day (CI: 8.18–10.23) and those who reported using cigarettes and other tobacco products reported 8.73 cigarettes per day (CI: 6.66–10.80). These mean values for these two groups were not significantly different as judged by overlapping 95% confidence intervals. The groups of nontobacco users and other tobacco products only also reported daily cigarette use at low levels: 1.52 cigarettes per day in the “not tobacco users” group and 1.69 cigarettes per day in the “other tobacco products only” group. Twenty-three percent (CI: 22%–25%) of the sample reported ever visiting a hookah bar or restaurant, 32% (CI: 29%–34%) of ever tobacco users reported trying their first tobacco product after age 18 and of those who became regular tobacco users, 39% (CI: 35%–43%) became a regular tobacco user after age 18.

In the multivariate model (Table 3), older young adults (aged 25–34) were significantly more likely to use cigarettes only or cigarettes and other tobacco products compared to those aged 18–24 (RRR = 1.48; CI: 1.07–2.06 and RRR = 1.60, CI: 1.03–2.49, respectively) and females were less likely to be dual users (RRR = 0.51; CI: 0.34–0.76) or to use other tobacco products only (RRR = 0.17; CI: 0.08–0.35) compared to males. Hispanics were less likely to use cigarettes or to be dual users and Blacks also had 61% reduced risk of other-tobacco product-only use compared to whites. Across all tobacco use categories, those with a Bachelor's degree or greater were significantly less likely to use tobacco products compared to those with some college education. Those with less than a high school education had a twofold increase in cigarette-only use (RRR = 2.42, CI: 1.53–3.83) and dual use (RRR = 2.00, CI: 1.05–3.81) compared to those with some college education. This pattern was similar for cigarette only use among those with a high school education compared to some college education (RRR = 2.06, CI: 1.44–2.95). Similar to the results from the bivariate analyses, individuals who reported that they “just meet” or “do not meet” basic expenses were more likely to use cigarettes only compared to those who reported “[meeting] needs with a little left” and participants reporting that they “do not meet” basic expenses were also twice as likely to be dual users (RRR = 2.06, CI: 1.03–4.14), after controlling for all other variables in the model.

## 4. Discussion

This study provides a unique focus on tobacco use patterns among young adults. It is the first paper in a series that presents baseline information on this population in the context of a longitudinal cohort designed to track the patterns, transitions, and trajectories of tobacco use behavior in this understudied age group. Young adults experience a significant developmental transition from living mostly at home or protected school environments to the freedoms and responsibilities of adulthood. Results of this study are intended to offer a clear understanding of tobacco product use prevalence in a young adult population and are reasonably consistent with national data, showing that more than half of the sample had ever smoked cigarettes and 19% of ever tobacco users aged 18–24 reported current cigarette use compared to the 20% national average for 18–24-year olds [2]. Findings from our study are also consistent with other recent studies which document the increasing prevalence of cigarette initiation after age of 18 and the high rates of transition to regular smoking in young adulthood [1, 22].

This study demonstrates a 30% dual use rate among current tobacco users, supporting previous studies indicating that 24–26% of young adult smokers are polytobacco users [22, 23]. It also shows that 64% of individuals who use other tobacco products smoke cigarettes concurrently. Interestingly, recent studies indicate that snus was introduced to test markets in 2006 [17], dissolvable tobacco products (including orbs, sticks, and strips) were introduced to test markets in 2008 [34], and some form of electronic cigarette has been on the market since at least 2006 [35]. The integration from test market to market suggests that the 4–6% increase in dual use found in this 2011 study as compared to the 2009 and 2010 data [22, 23] may be due to the increase in the array of available alternative tobacco products and/or tobacco company marketing efforts over time. In this study, dual users (cigarette smokers who also use one or more other tobacco products) report the same levels of smoking as cigarette-only users (8.73 cigarettes per day versus 9.20 cigarettes per day). This finding suggests that the use of other tobacco products does not replace cigarette smoking or decrease the mean number of cigarettes smoked daily among young adults. Additionally, the high prevalence of dip/snuff and chewing tobacco use among young adult cigarette smokers is consistent with a previous study showing high rates of smokeless tobacco and cigarette use among young males [26]. While a lower proportion of adults report dual use of smokeless tobacco and cigarettes in other national samples [26, 36], a longitudinal study showed that the quit rate was significantly lower for cigarette smoking compared to smokeless tobacco use and that there was little switching from cigarettes to smokeless tobacco in the USA (0.3% in one year) [36]. In a study of young adult military personnel, initiation of smokeless tobacco use was associated with harm escalation (i.e., smoking to dual use or smokeless to smoking or dual use) rather than harm reduction (i.e., smoking to smokeless only) [37]. Despite tobacco industry arguments that smokeless tobacco products provide a bridge to cessation [38], marketing of new smokeless tobacco products like snus in the USA encourages dual use by advertising these products as a substitute when cigarette smoking is unacceptable or prohibited [29]. Further, Camel Snus was test-marketed in some college communities, suggesting the targeting of these products for young adult smokers [29]. Our study confirms the high proportion of young adults reporting dual use of smokeless and combustible tobacco products, and supports concerns raised in previous studies about the role of smokeless tobacco use and dual use in smoking trajectories of young adults [26, 29, 37]. It also identifies differences in patterns of tobacco use and dual use by age, gender, race/ethnicity and socioeconomic status that could have long-term implications for tobacco-related health disparities.

Our study emphasizes the need for effective interventions to reduce the number of young adult smokers who progress from experimentation to regular use of tobacco products, change social norms about emerging tobacco products, and facilitate cessation of tobacco products in this age group. Recent studies suggest that media interventions may serve a key function in addressing all of these gaps [39], but these will need to be complemented with tailored and targeted strategies at the individual and community levels. Moreover, federal regulation of new tobacco products and their marketing also presents an unprecedented opportunity to reduce combusted cigarette and other forms of tobacco product consumption in this vulnerable age group via policy change and regulation of claims made by new and modified risk/reduced harm products and by use of targeted public education campaigns. In order to inform the regulatory process, rapid and reliable data will be needed [25]. This is especially important as new products using noncombustible forms of nicotine delivery are introduced that could have unintended consequences by delaying or negating cessation motivation or attracting new users, especially if the industry continues to target young adults by introducing appealing new products like ENDS, snus, and dissolvables into the marketplace [18].

### 4.1. Strengths/Limitations

This study harnesses the strengths of an existing online panel of adults to recruit a large, nationally-representative sample of young adults, a group typically identified as hard to reach. Smokers were oversampled for the purpose of this study in order to describe trajectories of cigarette, other tobacco product, and dual use in this population. Although the current analysis is limited to cross-sectional data from the baseline survey, future analyses will utilize longitudinal data to assess trends in young adult tobacco use over time. This study has several limitations: first, all tobacco product use is self-reported and may be subject to recall bias. The online nature of this panel study does not allow for biochemical validation of smoking status. Second, the survey was administered in English and Spanish and individuals who do not speak or are not literate in English or Spanish were unable to participate in this study. In addition, validity data is not available for the self-described financial situation measure. Finally, the small sample sizes for product use resulted in insufficient precision to report results for initiation of ENDS, snus, nicotine replacement products, and other tobacco products for certain population subgroups. This may be due to the overall low prevalence of use of these emerging products; thus, as emerging tobacco products gain attention in the marketplace, initiation with these products is likely to increase. We expect that future waves of data will have larger numbers of individuals initiating with emerging tobacco products as they gain popularity in the USA marketplace.

## 5. Conclusion

This study uses data from a large, nationally representative sample of young adults aged 18–34 to describe prevalence, patterns, and predictors of cigarette and dual use in this population. Of the 23% percent of young adults who were current tobacco users, 30% reported dual use. Similar levels of cigarette use were observed among cigarette-only users and dual users, indicating that dual use does not lead to harm reduction among young smokers. Further, nearly one-third of ever tobacco users in our study reported tobacco product initiation after the age of 18 and nearly 40% of regular users reported progressing to regular use during young adulthood. Due to the increased morbidity and mortality associated with tobacco use, disrupting transitions to regular smoking in young adults will result in tremendous benefits in terms of lives saved and disease prevented at the population level [40, 41]. This study highlights the need for improved monitoring of polytobacco use across the life course and development of tailored smoking prevention and cessation interventions for young adults. It also argues for the need to have rigorous but rapid surveillance in place to serve as an early warning sentinel system to inform regulation of new, emerging, and existing tobacco products by the FDA to protect the health of USA young adults [25]. Since smoking prevalence overall in the adult population (≥18 years) has stalled to around 20% in the past 5 years [42], interventions focused on the prevention and cessation of tobacco and polytobacco use in young adults can be critical to reversing the slowed decline in tobacco use among U.S. adults.

## Supplementary Material

Supplemental materials compare the demographic characteristics of young adults in Knowledge Networks' KnowledgePanel^®^ to the characteristics of young adult respondents to the Current Population Survey.

## Figures and Tables

**Table 1:** Prevalence of tobacco product by first use, ever use, and past 30-day use, using poststratification weights for the full sample.

	Ever use (*N* = 4201)		Tobacco product first used (*N* = 2493)		Mean age at initiation			Past 30-day use^a^			Mean no. of days used in past month^b^		
	Prevalence	95% CI	Prevalence	95% CI	*N*	Mean	95% CI	*N*	Prevalence	95% CI	*N*	Mean	95% CI
Cigarettes	0.51	(0.49–0.53)	0.73	(0.71–0.76)	1799	15.17	(14.94–15.40)	819	0.87	(0.83–0.90)	710	22.93	(21.76–24.10)
Cigars	0.31	(0.29–0.33)	0.11	(0.09–0.13)	283	17.19	(16.63–17.74)	666	0.19	(0.15–0.24)	132	5.88	(4.03–7.72)
Pipe (with tobacco)	0.09	(0.08–0.11)	0.01	(0.00–0.01)	15	16.34	(11.22–21.46)	629	0.05	(0.03–0.09)	24	10.14	(2.62–17.66)
Little cigars/cigarillos/bidis (like Black & Milds, Swisher Sweets, Phillies Blunt, or Captain Black)	0.26	(0.24–0.28)	0.05	(0.04–0.07)	158	16.92	(16.14–17.71)	648	0.16	(0.12–0.21)	117	10.52	(7.40–13.64)
E-cigarettes (like BLU or NJOY)	0.06	(0.05–0.07)	***	***	3	20.00	(14.51–25.43)	627	0.07	(0.05–0.11)	44	9.04	(4.38–13.69)
Chewing tobacco (like Levi Garrett, Red Man, or Beech Nut)	0.07	(0.06–0.09)	0.01	(0.01–0.02)	25	12.65	(10.18–15.11)	625	0.05	(0.03–0.09)	24	11.11	(4.88–17.34)
Dip/snuff (like Skoal or Copenhagen)	0.10	(0.09–0.11)	0.03	(0.02–0.04)	60	14.80	(13.73–15.88)	627	0.11	(0.08–0.16)	49	15.14	(9.77–20.51)
Snus (like Camel Snus)	0.06	(0.05–0.07)	***	***	3	19.25	(16.33–22.17)	626	0.05	(0.03–0.08)	36	10.96	(4.90–17.02)
Dissolvable tobacco products (like Ariva, Stonewall, Camel Orbs, Sticks or Strips)	0.01	(0.00–0.01)	—	—	—	—	—	619	0.01	(0.00–0.04)	7	16.29	(−4.87–37.46)
Hookah/shisha (hookah tobacco)	0.17	(0.16–0.19)	0.04	(0.03–0.05)	122	19.32	(18.50–20.15)	631	0.08	(0.05–0.12)	63	6.97	(1.97–11.96)
Nicotine products (like gum, patches, lozenges)	0.07	(0.06–0.08)	***	***	1	22	***						
Other^c^			***	***	3	17.85	(14.68–21.02)						
Unsure/decline to state			0.01	(0.00–0.02)	18	14.07	(9.61–18.52)						
refused			***	***									

*Note.* In all columns, *N* represents the unweighted denominator for prevalence estimates.

^a^Among those who report every day or someday use of cigarettes or other tobacco products.

^b^Among those who used at least one day in the past 30 days.

^c^All participants responding “other” identified “clove cigarettes” as the first product used.

***Insufficient precision to report.

—No responses.

**Table 2:** Demographics by current tobacco product use, using poststratification weights for the full sample (unweighted *N* = 4, 201).

	Not tobacco users		Cigarettes only		Cigarettes and othertobacco products		Other tobacco products only		
	Prevalence	95% CI	Prevalence	95% CI	Prevalence	95% CI	Prevalence	95% CI	*P* value
Overall	0.77	(0.75–0.79)	0.12	(0.11–0.14)	0.07	(0.06–0.08)	0.04	(0.03–0.05)	—
Age									0.129
18–24	0.78	(0.75–0.81)	0.11	(0.09–0.13)	0.06	(0.05–0.08)	0.05	(0.03–0.07)	
25–34	0.77	(0.74–0.79)	0.13	(0.11–0.15)	0.07	(0.06–0.09)	0.03	(0.02–0.04)	
Gender									<0.001
Male	0.75	(0.72–0.78)	0.10	(0.08–0.12)	0.09	(0.07–0.11)	0.06	(0.05–0.08)	
Female	0.80	(0.77–0.82)	0.14	(0.12–0.16)	0.05	(0.04–0.06)	0.01	(0.01–0.02)	
Race/ethnicity									0.017
White, non-Hispanic	0.75	(0.73–0.78)	0.13	(0.11–0.15)	0.07	(0.06–0.09)	0.04	(0.03–0.06)	
Black, non-Hispanic	0.75	(0.68–0.80)	0.13	(0.09–0.19)	0.11	(0.07–0.16)	0.02	(0.01–0.03)	
Other, non-Hispanic	0.84	(0.76–0.90)	0.10	(0.06–0.17)	0.03	(0.01–0.08)	0.03	(0.01–0.11)	
Hispanic	0.83	(0.79–0.87)	0.09	(0.06–0.12)	0.06	(0.04–0.08)	0.03	(0.01–0.05)	
Education									<0.001
Less than high school	0.68	(0.60–0.74)	0.20	(0.14–0.27)	0.12	(0.08–0.17)	0.01	(0.00–0.05)	
High school	0.68	(0.62–0.72)	0.19	(0.15–0.24)	0.09	(0.06–0.13)	0.04	(0.02–0.07)	
Some college	0.78	(0.75–0.81)	0.10	(0.08–0.12)	0.07	(0.05–0.09)	0.05	(0.04–0.07)	
Bachelor's degree	0.89	(0.86–0.92)	0.05	(0.03–0.08)	0.03	(0.02–0.05)	0.02	(0.01–0.05)	
Graduate or professional degree	0.93	(0.90–0.96)	0.04	(0.02–0.08)	0.02	(0.01–0.04)	0.01	(0.00–0.01)	
Current employment status									0.218
Work full-time (35 hours/week or more)	0.78	(0.75–0.81)	0.11	(0.09–0.13)	0.07	(0.05–0.09)	0.05	(0.03–0.06)	
Work part-time (15–34 hours/week)	0.78	(0.73–0.83)	0.11	(0.08–0.15)	0.07	(0.05–0.10)	0.04	(0.02–0.07)	
Work part-time (less than 15 hours/week)	0.70	(0.61–0.78)	0.16	(0.11–0.23)	0.11	(0.06–0.21)	0.02	(0.01–0.09)	
Do not currently work for pay	0.77	(0.74–0.81)	0.13	(0.11–0.17)	0.07	(0.05–0.09)	0.02	(0.01–0.04)	
Financial situation									<0.001
Live comfortably	0.86	(0.82–0.89)	0.05	(0.04–0.08)	0.04	(0.03–0.07)	0.04	(0.03–0.07)	
Meet needs with a little left	0.81	(0.78–0.84)	0.10	(0.08–0.12)	0.06	(0.04–0.08)	0.03	(0.02–0.05)	
Just meet basic expenses	0.72	(0.69–0.76)	0.16	(0.13–0.19)	0.08	(0.06–0.11)	0.04	(0.02–0.06)	
Do not meet basic expenses	0.63	(0.55–0.71)	0.23	(0.17–0.32)	0.12	(0.08–0.19)	0.01	(0.01–0.03)	

**Table 3:** Relative risk ratios (RRRs)^1^ of tobacco product use compared to no tobacco use (weighted *N* = 4, 157).

	Cigarettes-only versus no tobacco use		Cigarettes and other tobacco products versus no tobacco use		Other tobacco products only versus no tobacco use	
	RRR	(95% CI)	RRR	(95% CI)	RRR	(95% CI)
Age						
18–24	Ref.		Ref.		Ref.	
25–34	1.48	(1.07–2.06)*	1.60	(1.03–2.49)*	0.84	(0.47–1.50)
Gender						
Male	Ref.		Ref.		Ref.	
Female	1.29	(0.96–1.73)	0.51	(0.34–0.76)**	0.17	(0.08–0.35)**
Race/ethnicity						
White, non-Hispanic	Ref.		Ref.		Ref.	
Black, non-Hispanic	0.74	(0.45–1.20)	1.06	(0.60–1.90)	0.39	(0.20–0.78)*
Other, non-Hispanic	0.84	(0.44–1.61)	0.42	(0.14–1.25)	0.67	(0.14–3.27)
Hispanic	0.38	(0.25–0.59)**	0.45	(0.25–0.79)*	0.56	(0.26–1.23)
Education						
Less than high school	2.42	(1.53–3.83)**	2.00	(1.05–3.81)*	0.24	(0.04–1.47)
High school	2.06	(1.44–2.95)**	1.41	(0.87–2.29)	1.04	(0.52–2.08)
Some college	Ref.		Ref.		Ref.	
Bachelor's degree	0.42	(0.25–0.73)*	0.34	(0.16–0.68)*	0.27	(0.12–0.58)**
Graduate or professional degree	0.32	(0.16–0.63)**	0.19	(0.07–0.51)**	0.09	(0.03–0.26)**
Current employment status						
Work full time (35 hours/week or more)	Ref.		Ref.		Ref.	
Work part time (15–34 hours/week)	0.78	(0.48–1.24)	1.02	(0.57–1.85)	0.88	(0.42–1.87)
Work part time (less than 15 hours/week)	1.18	(0.70–1.99)	1.74	(0.80–3.81)	0.56	(0.13–2.46)
Do not currently work for pay	0.71	(0.50–1.01)	0.87	(0.52–1.45)	0.71	(0.35–1.42)
Financial situation						
Live comfortably	0.61	(0.38–0.98)*	0.80	(0.44–1.46)	1.26	(0.63–2.49)
Meet needs with a little left	Ref.		Ref.		Ref.	
Just meet basic expenses	1.67	(1.20–2.33)*	1.25	(0.76–2.08)	1.15	(0.57–2.33)
Do not meet basic expenses	2.79	(1.72–4.51)**	2.06	(1.03–4.14)*	0.65	(0.22–1.90)

*P < 0.05, **P < 0.001.

^1^Relative risk ratios were calculated using multinomial logistic regression and are adjusted for survey weights and all other variables in the model.
