# Supplementary material for: Patterns of Tobacco Use and Dual Use in US Young Adults: The Missing Link between Youth Prevention and Adult Cessation
Source: J Environ Public Health. 2012 May 14;2012:679134. doi: 10.1155/2012/679134 (PMC3361253; doi:10.1155/2012/679134)
Supplement: Supplementary file 1 — Supplemental materials compare the demographic characteristics of young adults in Knowledge Networks' KnowledgePanel® to the characteristics of young adult respondents to the Current Population Survey. [file 679134.f1.pdf]

Appendix A: KnowledgePanel® Demographic Comparisons – 18-34 Year Olds – August 2011

		Adult Panel Members[1]	Adult U.S. Population (June 2011 CPS[2] except as footnoted)
**Gender**	Male	49.5%	50.3%
	Female	50.5%	49.7%
**Age**	18–24	41.9%	41.8%
	25–34	58.1%	58.2%
**Race**	White Only	73.4%	77.3%
	Black (African American) Only	15.8%	14.2%
	American Indian, Alaskan Native Only	0.8%	1.0%
	Asian Only	3.4%	5.1%
	Hawaiian or Pacific Islander Only	0.8%	0.3%
	2+ Races	5.8%	2.1%
**Hispanic Ethnicity**	Hispanic	19.7%	19.5%
	Non-Hispanic	80.3%	80.5%
**Employment Status**	In the Labor Force	84.2%	75.7%
	Employed	62.5%	66.3%
	Unemployed	21.7%	9.4%
	Not in the Labor Force	15.8%	24.3%
**Marital Status**	Married	34.2%	30.6%
	Not Married	65.8%	69.4%
**Housing Ownership[3]**	Own	53.7%	53.4%
	Rent/Other	46.3%	46.6%
**Level of Education**	Less than High School Diploma	15.6%	14.5%
	High School Diploma or Equivalent	30.3%	28.4%
	Some College	31.6%	33.9%
	Bachelor's Degree or Beyond	22.6%	23.3%
**Household Income[4]**	Under $10,000	7.3%	8.4%
	$10,000–$24,999	16.0%	17.1%
	$25,000–$49,999	25.8%	27.4%
	$50,000–$74,999	17.2%	19.6%
	$75,000 or more	33.7%	27.5%
**Census Region**	Northeast	16.7%	17.7%
	Midwest	21.1%	21.2%
	South	37.2%	36.8%
	West	25.1%	24.3%
**Internet Access (Household)[5]**	Any Connection Speed	75.6%	78.2%
	Broadband	73.1%	76.8%

When you are making a marketing investment decision or conducting social science research, only KnowledgePanel® will do!

[1] Active profiled adults are weighted to be representative of the U.S. population on age, gender, race, Hispanic ethnicity, language proficiency, region, metro status, education, household income, homeownership, and Internet access using post-stratification adjustments to offset any non-response or non-coverage bias.

[2] Estimates were calculated using June 2011 CPS microdata available at *www.census.gov*. The data are weighted using CPS final individual weights.

[3] National housing statistics are from March 2010 CPS Annual Social and Economic Supplement.

[4] National income statistics are from March 2010 CPS Annual Social and Economic Supplement.

[5] National Internet coverage statistics are from October 2010 CPS Internet and Computer Usage Supplement.

© 2011 Knowledge Networks, Inc.
